# Kidney surface development in human fetuses: study applied to radiological diagnosis

**DOI:** 10.1590/S1677-5538.IBJU.2022.9977

**Published:** 2022-08-20

**Authors:** Luciano A. Favorito, Marcio Luiz P. Lobo, Ana Vitória Fernandes, Carla M. Gallo, Francisco J. B. Sampaio

**Affiliations:** 1 Universidade do Estado do Rio de Janeiro Unidade de Pesquisa Urogenital Rio de Janeiro RJ Brasil Unidade de Pesquisa Urogenital - Universidade do Estado do Rio de Janeiro – Uerj, Rio de Janeiro, RJ, Brasil

**Keywords:** Kidney, Branchio-Oto-Renal Syndrome, Embryology

## Abstract

**Objective:**

To evaluate the anatomical aspects of the kidney surface in human fetuses during the second gestational trimester.

**Material and Methods:**

We studied 108 kidneys obtained from 54 human fetuses (29 males and 25 females). The kidney was dissected and the number of clefts was counted. The renal volume was also assessed. To compare the quantitative data in both sexes, the Students-t-test was used (p < 0.05). Simple linear correlations were calculated for all kidney measurements, according to fetal age. Statistical analysis was performed with the R program (Version 3.5.1).

**Results:**

The fetuses ranged in age between 11.4 to 23 weeks post-conception. The renal volume of the right kidney ranged from 0.09 to 2.397 cm (mean=0.8479) and the renal volume of the left kidney ranged from 0.07 to 2.416 cm (mean=0.8036). The mean number of renal clefts in fetuses studied was 15.25 (7 to 28). There was no statistical significant difference in renal clefts between the sides either in males (p = 0.646) or in females (p = 0.698). Also, there was no significant difference in the mean number of renal clefts between male and female fetuses in right kidney (p = 0.948) and in left kidney (p = 0.939).

**Conclusions:**

The number of renal clefts has a great variation, weak correlation and no tendency to decrease during the 2nd gestational trimester. The number of clefts in right kidney of total sample and female fetuses has a significant development with age.

## INTRODUCTION

The 2nd gestational trimester is very important for the embryonic development of the kidneys, renal pelvis, ureter and bladder (1, 2). An important branching of the ureteric bud occurs between the 5th and 14th weeks post conception, leading to formation of the major and minor renal calyces, renal pelvis and collecting tubules (3, 4). This branching will be important to the renal lobulations development.

The surface of the fetal kidney is divided by a number of clefts into lobes and lobules. Fetal kidney lobes (clefts) are fine, linear demarcations indenting the renal surface, separating normal lobes, consisting of a central pyramid, and surrounding cortex (5, 6). The interlobular boundary lines are apparent as grooves on the surface of the fetal kidney but are rarely visible in the mature kidney (7). The persistent fetal kidney lobulation is a rare anatomic variant and can mimic a renal neoplasm leading to a wrong radiological diagnosis. This condition is denominated as renal pseudotumors (8).

Studies of the renal clefts development in human fetuses are rare. We hypothesized that the renal clefts are observed during the 2nd gestational trimester without differences between the genders and sides and showing a lower incidence at the end of this period. The objective of this work is to evaluate the anatomical aspects of the kidney surface in human fetuses during the 2nd gestational trimester.

## MATERIALS AND METHODS

The study was approved according to the ethical standards of the hospital's institutional committee on experimentation with human beings (IRB: 4.088.773, CAAE:31780419.0.0000.5259).

We studied 108 kidneys obtained from 54 human fetuses (29 males and 25 females) ranging in age from 11.4 to 23 weeks post-conception (WPC). The fetuses were macroscopically well preserved, with no signs of malformation, and the stillbirth was due to hypoxia. Gestational age was determined at WPC according to the length of the foot. Currently, this criterion is considered the most acceptable parameter for estimating gestational age (9–11). The fetuses were also evaluated regarding total length (TL), crown-rump length (CRL) and body weight immediately before dissection with the aid of a digital pachymeter. The same observer made all the measurements (12, 13). The fetuses were donated by the hospital's obstetrics department. All data were collected from July 2018 to November 2021.

**Figure 1:**
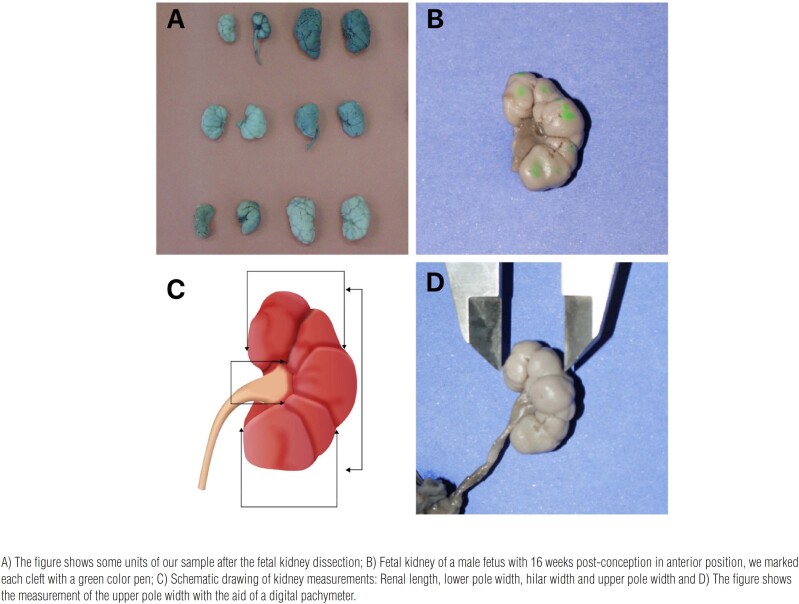
Renal clefts and kidney measurements.

Using a standardized technique, the fetuses were carefully dissected with extraction of the kidneys and ureters with the aid of a stereoscopic lens with 16/25X magnification. All fetuses were dissected under identical conditions by the same researcher, who has practical experience in microsurgery. After kidney dissection, we evaluated the following measurements with the aid of a digital pachymeter: renal length, width of the superior pole, width of the inferior pole, and renal thickness (Figure-1). The data were expressed in centimeters. The fetal renal volume was calculated using the ellipsoid formula (14): Renal volume (RV) = [renal length x renal thickness x renal width (lower pole + upper pole)/2] x 0.523.

After the measurements the kidney was dissected in our laboratory with the aid of a microscope (Zeiss Discovery V8 microscope with stereoscopic lens with 16/25X magnification) and the number of clefts was counted (Figure-1). We observed the fetal kidneys in anterior position, lateral position, and posterior position and to facilitate the clefts number identification we marked each cleft with a color pen (Figure-1).

### Statistical Analysis

All parameters were statistically processed and graphically described. To compare the quantitative data in both sexes, the students-t-test was used (p < 0.05). Simple linear correlations (where r² values less than 0.4 reflect very weak correlation, r² between 0.4 and 0.7 reflect moderate correlation and r² greater than 0.7 indicate strong correlation) were calculated for all kidney measurements, according to fetal age. The statistical analysis was performed with the R program (Version 3.5.1).

## RESULTS

The fetuses ranged in age between 11.4 to 23 WPC, weighted between 60 and 780g, and had crown-rump length between 7.3 and 22.2 cm. The mean number of renal clefts in fetuses studied was 15.25 (7 to 28). The statistical analysis of all kidneys biometric parameters measurements is reported in Table-1.

**Table 1:** The table presents the statistical analysis of the main fetal parameters and the renal clefts in 54 fetuses studied.

	GENDER																	
	MALE						FEMALE						TOTAL					
	n	Mean	Median	SD	Min	Max	n	Mean	Median	SD	Min	Max	n	Mean	Median	SD	Min	Max
Age (WPC)	29	16.628	16.60	2.327	11.40	23.00	25	17.304	17.40	1.680	13.00	20.40	54	16.941	17.00	2.063	11.40	23.00
Total length (cm)	29	22.841	23.00	3.528	15.00	32.00	25	23.480	24.50	3.100	13.50	28.00	54	23.137	23.50	3.321	13.50	32.00
Weight (g)	29	261.207	245.00	133.568	60.00	780.00	25	275.600	285.00	102.045	60.00	455.00	54	267.870	270.00	119.134	60.00	780.00
N° of Clefts (R)	29	15.207	15.00	4.924	7.00	26.00	25	15.120	14.00	4.711	10.00	27.00	54	15.167	14.00	4.781	7.00	27.00
N° of Clefts (L)	29	15.379	15.00	4.648	7.00	26.00	25	15.280	14.00	4.792	7.00	28.00	54	15.333	14.50	4.670	7.00	28.00
Vol. RK (cm)	26	0.7887	0.6640	0.5099	0.2340	2.3970	23	0.9148	0.8300	0.4853	0.0900	2.1600	49	0.8479	0.7080	0.4974	0.0900	2.3970
Vol. LK (cm)	26	0.7399	0.5995	0.4946	0.1630	2.4160	23	0.8757	0.7800	0.5316	0.0700	2.4100	49	0.8036	0.6570	0.5115	0.0700	2.4160
CRL (cm)	29	14.659	15.00	3.399	7.30	22.20	25	15.884	16.00	2.059	9.50	19.30	54	15.226	15.75	2.899	7.30	22.20

**SD** = Standard Deviation; **WPC**= weeks post conception; **cm** = centimeters; **RK** = right; **LK** = left kidney; **CRL** = Crown rump length.

There was no statistically significant difference in renal clefts between the sides either in males (p = 0.646) or in females (p = 0.698). Also, there was no significant difference in the mean number of renal clefts between male and female fetuses in right kidney (p = 0.948) and in left kidney (p = 0.939). The renal volume of the right kidney ranged from 0.09 to 2.397 cm (mean = 0.8479) and the renal volume of the left Kidney ranged from 0.07 to 2.416 cm (mean = 0.8036). The analysis of the fetal kidney volume showed no significant statistical difference between side and sex comparisons (Volume of right kidney x left kidney (males and females): p = 0.057; Volume of right kidney x left kidney (males): p = 0.067; Volume of right kidney x left kidney (females): p = 0.333; Males x females (volume of right kidney): p = 0.381 and Males x females (volume of left kidney): p = 0.359).

The linear correlation was performed to enable analysis of morphological data at different gestational ages. Results for male fetuses’ renal volume (Right kidney: r = 0.763, p < 0.001 and Left kidney: r = 0.755, p < 0.001) and female fetuses’ renal volume (Right kidney: r = 0.698, p < 0.001 and Left kidney: r = 0.751, p < 0.001), indicated that the renal volume increased significantly during the fetal period studied in males and females fetuses.

**Figure 2:**
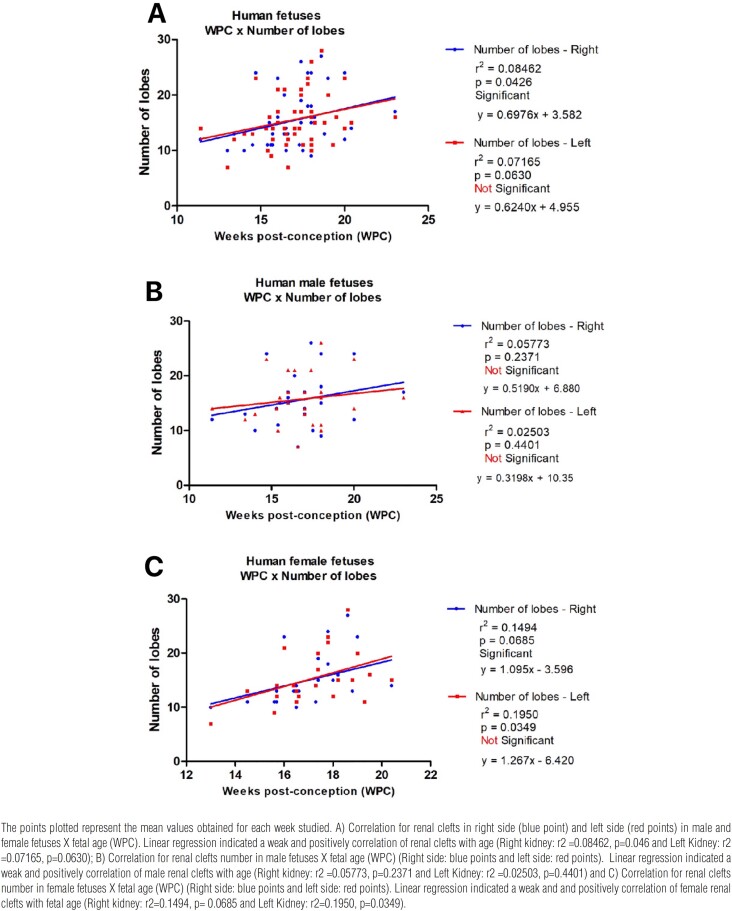
Linear regression analysis comparing the correlation of the renal clefts and fetal age (WPC).

The linear correlation of renal lobes was performed to enable analysis of morphological data at different gestational ages. The linear correlation of the 54 fetuses renal clefts is shown in Figure-2. The linear correlation indicated that the fetal renal lobes number increased with age both in total sample, female and male fetuses, but the differences were not statistically significant, and the correlation was weak. The exception was the renal right clefts in total sample and female fetuses that has a significant development with age.

## DISCUSSION

In the beginning of the 5th week of development, the ureteral buds originate at the distal portion of the mesonephric ducts and merges with the metanephrogenic blastema (15, 16). In this paper we studied fetuses of the 2nd trimester of gestation. At this age, the kidneys are expected to have reached their final position and from then on they will only develop in size, representing an ideal moment for this study. In our sample we observed that the renal parenchymal volume had a strong and positive correlation with fetal age during the 2nd gestational trimester.

The branching of the ureteral buds will determine the pyelocaliceal pattern and the corresponding renal lobules (2, 17, 18). The kidney fetal lobe is made up of medullary pyramid surrounded by cortex, separated by interlobar grooves and drained by single calyx. The kidney interlobar grooves disappear during the third gestational trimester (5).

The persistence of kidney fetal clefts is a rare anatomic variant characterized by fine, linear demarcations indenting the renal surface between normal renal lobes and consisting of normal central pyramids and surrounding cortex which are mistaken as tumor and can lead a difficulty radiological diagnosis. This condition is called renal pseudotumor (8, 19). Renal pseudotumor is a term that include persistent fetal lobulation, hypertrophy of Bertin columns and dromedary humps (8, 19).

Radiological confirmation of persistent fetal lobulation of kidney can be made by documenting the presence of renal pyramid in the bulge bounded by septa of Bertin on either side (20). Radiologist can make potential errors during image interpretation of persistent fetal lobulation especially on conventional and power Doppler ultrasound scan and to reach a secure diagnosis, a CT or MRI should be done (7).

The fetal kidney grooves become invisible during the third trimester resulting in smooth renal surface (5, 21). This paper presents the first normative parameters of fetal renal clefts development during the 2nd gestational trimester, and we observed that the renal clefts number increased with age, however the correlation was weak in the fetal period analyzed, except in the right side of total sample and in the right side of female fetuses where the number of clefts has a significant development with age. We observed that the differences of the development of renal clefts number were not statistically significant between genders and side.

Renal clefts showing no tendency to decrease during the 2nd gestational trimester and this finding may justify the persistence of renal lobulations during the 3rd gestational trimester and after birth, however the absence of 3rd gestational trimester fetuses in our sample to determine the moment when the lobulations begin to disappear is an important limitation of this work.

## CONCLUSIONS

The number of renal clefts has a great variation, weak correlation, and no tendency to decrease during the 2nd gestational trimester. The number of clefts in right kidney of total sample and female fetuses has a significant development with age.
